# Tuning RGD Motif and Hyaluronan Density to Study Integrin Binding

**DOI:** 10.3389/fphys.2018.01022

**Published:** 2018-08-07

**Authors:** Cornelia Zapp, Burcu B. Minsky, Heike Boehm

**Affiliations:** ^1^Department of Cellular Biophysics, Max Planck Institute for Medical Research, Heidelberg, Germany; ^2^Physical Chemistry, Heidelberg University, Heidelberg, Germany

**Keywords:** QCM-D, hyaluronan, integrins, cell adhesion, proteoliposomes

## Abstract

Well-controlled surfaces with immobilized substrates enable novel approaches to investigate specific aspects of biological processes related to cell adhesion or motility. A subset of integrins, cellular transmembrane glycoproteins, recognize the evolutionarily conserved tripeptide sequence RGD, and anchor cells to their surrounding proteins as well as mediate bidirectional signaling. In this study, the main question was how co-presentation of hyaluronan (HA), an essential component of the extracellular matrix (ECM), and the RGD motif affect integrin binding. We report a method to prepare self-assembled monolayers on gold surfaces, co-presenting the cell adhesive RGD motif and small HA molecules, to investigate integrin containing proteoliposome binding. This technique enables an independent adjustment of the RGD motif and HA density while maintaining a passivating background: Layer formation and subsequent interactions with α_IIb_β_3_ integrins, which are reconstituted in liposomes, was monitored by label-free quartz crystal microbalance with dissipation monitoring (QCM-D). Exceeding a critical RGD motif density of 40% results in enhanced binding of proteoliposomes. Co-presentation studies with varying HA and constant RGD motif density demonstrate that marginal amounts of HA are sufficient to prevent integrin binding. These findings are of specific importance in relation to cancer cell microenvironments, which show highly enriched HA in the surrounding ECM to reduce adhesion properties.

## Introduction

Nature has generated well-adapted processes and materials through natural selection. Thus, nature in its remarkable diversity provides an extraordinary collection of strategies. These can be exploited as a source of inspiration and adopted for biological applications. As such, the interdisciplinary field of biomimetics research encompasses many specialty areas in biology, materials science, and nanotechnology. The fundamental idea is to generate synthetic systems to mimic biological concepts or to accomplish biological tasks. One approach to utilize this synthetic biological concept is the “bottom-up” approach, in which a minimal number of synthetic or biological molecules are combined to realize a desired function (Tu and Tirrell, 2004; Benner and Sismour, 2005; Purnick and Weiss, 2009). Molecular biomaterials can be fabricated in a “bottom-up" approach, in which molecules adapt a defined arrangement and consequently produce novel macromolecular assemblies. Molecular assembly can be applied to modulate surface properties of a material’s interface, where chemical, physical, and biological processes are maintained. Surfaces are of foremost importance in biological systems, since surface-cell interactions modulate how cells attach to the material. Specifically modified surfaces may serve as a research platform to study specific aspects of cell interaction.

The extracellular matrix (ECM) is a complex dynamic meshwork consisting of diverse macromolecules, which are secreted by the embedded cells within the tissue and provide biochemical and structural support. Cells sense chemical, topographic and mechanical features via specific cellular receptors and mediate the assembling of adhesion complexes to transfer information via signaling pathways (Geiger and Yamada, 2011; Jansen et al., 2017). Thus, cell adhesion to the surrounding matrix and neighboring cells plays a key role in many cellular processes including survival, differentiation and proliferation (Berrier and Yamada, 2007). Integrins are the most abundant and fundamental matrix receptors on animal cells (Mecham, 2011). These transmembrane heterodimeric glycoproteins fulfill two main functions: (1) attachment of cells to the ECM and (2) inducing bidirectional signal transduction between the ECM and cells (Streuli, 2016; Gauthier and Roca-Cusachs, 2018). A prevalently surface-displayed and evolutionarily conserved RGD motif consists of the three amino acids arginine, glycine, and aspartate. The RGD motif is present in various proteins of the ECM, for instance fibronectin, vitronectin, and fibrinogen (Mecham, 2011). A subset of integrins specifically recognize RGD repeats to anchor cells to the ECM and mediate bidirectional signaling. Therefore, studying cell adhesion demands precise control of RGD immobilization on the artificial surfaces to focus mainly on the surface properties required for cellular interactions via integrins and prevent non-specific protein attachment.

The non-sulfated glycosaminoglycan hyaluronan (HA) is a major non-proteinaceous component of the ECM and is formed of repeating disaccharide units of glucuronic acid and *N*-acetyl glucosamine. HA provides structural support, maintains tissue hydration, and serves as a lubricant in certain tissues (Dicker et al., 2014). HA interacts with a large number of HA-binding proteins (hyaladherins), matrix components and cells. Its temporal and spatial distribution as well as size plays critical roles in numerous biological processes, e.g., wound healing, inflammation and tumor progression (Dicker et al., 2014). Although HA is an essential component of extracellular structures and is reported to contribute to diverse cellular functions, little is known about its role in cellular adhesion processes. It has been reported, that HA can have both adhesive and anti-adhesive properties as well as promote cell detachment (Evanko et al., 2007; Cao et al., 2009). Moreover, showing that inhibition of CD44 on hematopoietic progenitor cells prevents rolling and adhesion to an HA-coated surface (Hanke et al., 2014) or that HA mediates also early, long-ranging adhesive interactions between cells and the surrounding surface, which precede integrin-mediated adhesion and formation of focal adhesions (Zimmerman et al., 2002; Zaidel-Bar et al., 2004) demonstrates the importance of HA specific interactions. In order to understand the role of HA in the dynamic process of cell adhesion, we aim to establish a synthetic model with reduced complexity, giving us the opportunity to focus on the repulsive forces of HA.

Numerous studies have applied the chemical functionalization of surfaces with desired properties to study the effect on cell structures, metabolism, viability or proliferation among others (Yao et al., 2013; Jeon et al., 2014; Ventre and Netti, 2016). One approach for the chemical functionalization of surfaces is the formation of self-assembled monolayers (SAMs) on gold surfaces through thiol residues (Love et al., 2005). Thereby precise control over the properties of the designed biological interfaces can be exerted by the molecular structure and the surrounding environment.

Alkanethiols terminated with oligo(ethylene glycol) moieties (OEG-alkanethiols) form SAMs on gold surfaces, which are resistant to unspecific protein adsorption. These surfaces have been exploited in several studies including cell adhesion or stem cell differentiation (Tidwell et al., 1997; Arima and Iwata, 2015; Hao et al., 2016; Almeida and Shukla, 2017).

The well-defined surfaces employed in this study were prepared according to the protocol published by Minsky et al. (2016), in which a controlled immobilization strategy in a two-layer system, simultaneously prevents unspecific protein binding and enables adjusting of the immobilization density on a surface. For this purpose SAMs are formed spontaneously on gold surfaces by co-adsorption of two functionalized oligo(ethylene glycol)-alkanethiols and unfunctionalized OEG-alkanethiols. By tuning the ratio of the two components, the density of the surface-displayed motif can be defined while a passivating background is maintained.

Work in our lab has focused on designing a bioinspired and well-controlled artificial integrin adhesion model system, in which surfaces, including the RGD binding motif and/or HA on an otherwise passivating background layer represent ECM mimetic, and reconstituted transmembrane integrins within lipid vesicles represents cell mimetic. Integrins form non-covalently associated heterodimeric complexes of an α- and a β-subunit, each having a large extracellular domain, a membrane spanning region and a short cytoplasmic domain (Hynes, 2002; Barczyk et al., 2010). In order to resemble the natural arrangement, isolated α_IIb_β_3_ integrins were reconstituted into liposomes, built up of amphiphilic molecules forming phospholipid bilayers and surrounding an aqueous unit (Akbarzadeh et al., 2013).

Within this study large unilamellar liposomes and liposomes with reconstituted α_IIb_β_3_ integrin (hereinafter referred to as proteoliposomes) were prepared by detergent removal (Erb et al., 1997). The aim of this study is to address the question of how co-presentation of HA and the RGD motif in varying densities impacts the attachment of integrins.

## Materials and Methods

### Chemicals

Purified L-α-phosphatidylcholine (eggPC) and L-α-phosphatidyl-DL-glycerol (eggPG) were purchased from Avanti Lipids Polar Inc. (Alabaster, AL, United States). Biobeads SM-2 (20–50 mesh size) were obtained from Bio-Rad Laboratories Inc. (Hercules, CA, United States). Triton X-100, sodium cyanoborohydride, and propargylamine were purchased from Sigma-Aldrich (Steinheim, Germany). Hyaluronic acid sodium salt (M_r_ = 10 kDa, sHA) was procured from LifeCore Biomedial (Chaska, MN, United States). HS–(CH)_11_–EG_3_–OH (EG_3_OH) and HS–(CH)_11_–EG_6_–N_3_ (EG_6_N_3_) were obtained from Prochimia (Sopot, Poland). Alkylated RGD peptide (sequence: GRGDSP) was purchased from Peptide Specialty Laboratories GmbH (Heidelberg, Germany). Dialysis tube (MWCO: 3,500 Da) was purchased from VWR (Radnor, PA, United States). Integrin α_IIb_β_3_ was extracted from outdated human blood platelets obtained by the Red Cross Germany, according to the protocol from Müller et al. with modifications (Müller et al., 1993; Hu et al., 2000).

### Proteoliposome Preparation by Detergent Removal

The protein was reconstituted into proteoliposomes according to the general procedure developed by Erb et al. (1997). Typically, an equimolar lipid mixture of eggPC/eggPG (0.43 μmol each) was vacuum – dried and resuspended in 1 mL buffer B (20 mM TRIS, 50 mM NaCl, 1 mM CaCl_2_, and 0.1% Triton X-100 at pH 7.4). For the preparation of proteoliposomes, the lipids were solubilized in 827 μL buffer B and 174 μL integrin α_IIb_β_3_ (0.23 mg/mL) was added to a final protein:lipid ratio of 1:5,000. Afterwards, the mixture was incubated at 37°C for 2 h under shaking. The detergent Triton X-100 was removed with 50 mg Bio-Beads SM-2 under stirring for 3.5 h. After removing the Bio-Beads SM-2, the detergent removal step was repeated. The obtained solution was stored at 4°C for a total maximum of 24 h. Before usage the resulting vesicles were processed by extrusion with a nominal 100 nm pore membrane (Whatman, Maidstone, United Kingdom) 11 times using a mini-extruder apparatus (Avanti Polar Lipids Inc., Alabaster, AL, United States). DLS measurements show an average size of 137.7 ± 41.9 nm for pure liposomes and 162.1 ± 41.7 nm for proteoliposomes. Vesicles were generally prepared at a nominal lipid concentration of ∼0.675 mg/mL, and then diluted 1:20 in 20× activation buffer (1× activation buffer: 50 mM NaCl, 20 mM TRIS, 1 mM CaCl_2_, 1 mM MgCl_2_, and 1 mM MnCl_2_, pH 7.4) before the experiment.

### Sample Preparation for Formation of a Functionalized Self-Assembled Monolayer

Click reaction was performed using TRIS buffer (100 mM, pH 8.5), ascorbic acid (100 mM), EG_6_N_3_ (150 μM) and alkylated RGD peptide sequence (480 μM) in the presence of 1 mM CuSO_4_. After 1.5 h at RT the reaction was stopped by adding EDTA (1 mM) to chelate copper. Mixtures with different ratios were prepared by diluting EG_6_RGD with EG_3_OH and adjusted to a total thiol concentration of 100 μM in passivation buffer. Surfaces were prepared by immobilization of this OEG mixture on the gold surface of the quartz crystal microbalance with dissipation monitoring (QCM-D) sensor, followed by a final washing step in passivation buffer.

### Quartz Crystal Microbalance With Dissipation Monitoring Measurements

For QCM-D experiments a fully automated Q-sense Omega Auto instrument (Biolin Scientific AB, Västra Frölunda, Sweden) with gold-coated quartz crystal electrodes (QSX301, Q-sense, AT-cut, 4.95 MHz, Biolin Scientific AB, Västra Frölunda, Sweden) was used. The system was operated in flow mode with a flow rate of 20 μL/min at 24°C. Frequency and dissipation data were collected from six overtones (*n* = 3, 5, 7, 9, 11, 13). Prior to use the electrodes were cleaned in a 5:1:1 solution of water, 30% hydrogen peroxide and 25% ammonia at 75°C for 10 min and activated in an UV/Ozone cleaner (ProCleaner, Bioforce Nanosciences, Ames, IA, United States) for 10 min. The Omega Auto includes a fully automated sample handling of four sensors in parallel and each sequence of an experiment was performed in duplicates. Changes in dissipation and normalized frequency, Δ*f* = Δ*fn*/*n*, of the seventh overtone (*n* = 7) are presented in the graphs. Frequency and dissipation changes were calculated by averaging over the last 5 min of the buffer wash before adding the samples and the final buffer wash. Buffers used as baselines were passivation buffer (50 mM NaCl, 20 mM TRIS, 1 mM CaCl_2_, and 1 mM MgCl_2_, pH 7.4), activation buffer (passivation buffer with 1 mM MnCl_2_, pH 7.4), and TRIS buffer (100 mM, pH 8.5).

### Synthesis of End-Alkylated Hyaluronan

Functionalization at the reducing *N*-acetylglucosamine unit of sHA was performed according to the protocol established by Lee et al. (2008). In short, 100 mg sHA and propargylamine (523 mM) were dissolved in borate buffer (20 mL, 100 mM, pH 8.5) containing sodium chloride (400 mM) for 2 h at RT. After adding sodium cyanoborohydride (200 mM) the solution was stirred for 5 d at 40°C. The mixture was dialyzed once against water, containing sodium chloride and hydrochloric acid, followed by water containing hydrochloric acid for 2 d. Prior to storage at −20°C the end-alkylated sHA was lyophilized.

### Preparation of a Dual-Functionalized Self-Assembled Monolayer

For preparing the dual functionalized surfaces, a constant ratio of EG_6_RGD (40%) was mixed with EG_3_OH and EG_6_N_3_ (0–100% EG_6_N_3_). After immobilization of this OEG mixture on the gold surface of the QCM-D sensor, end-alkylated HA (1.875 mg/mL) was conjugated to the EG_6_N_3_ in the presence of ascorbic acid (100 mM) and CuSO4 (1 mM) in TRIS buffer (100 mM, pH 8.5). Finally, the surface was washed with TRIS buffer to remove unbound molecules.

## Results and Discussion

**FIGURE 1:**
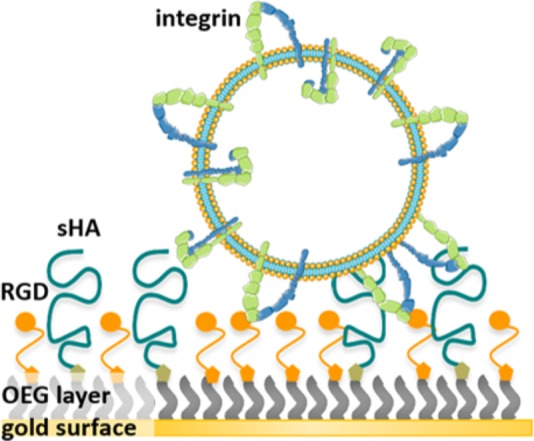
Illustration of the working principle: First, a dual-functionalized SAM is prepared enabling independent adjustment of the sHA density and RGD binding motif density, respectively. Second, the binding of integrins reconstituted in liposomes to the RGD binding motif within these surfaces was assessed.

Within this study dual-functionalized surfaces were prepared by co-presenting the RGD motif and HA in varying densities on a passivating background. The average contour length of the short HA (sHA < 10 kDa) is around 24 nm and its radius of gyration is around 5.3 nm (Takahashi et al., 1999; Buhler and Boué, 2004). A mixture of OEGs, containing OEG-alkanethiol (EG_3_OH), OEG-alkanethiols functionalized with RGD motif (EG_6_RGD), and OEG-alkanethiols with a terminal azide group (EG_6_N_3_), were immobilized on the gold surface. Subsequently, end-alkylated HA was conjugated to the OEG-azide group in the immobilized OEG layer directly on the surface via an *in situ* azide/alkyne cycloaddition reaction. This extended surface preparation approach facilitates the variation of the HA density and RGD density independently of each other, while a passivating background is maintained. **Figure 1** illustrates the working principle. Following the surface preparation, the attachment of liposomes and proteoliposomes on these well-defined surfaces was monitored using QCM-D. QCM-D is a label-free, real-time measurement technique for monitoring molecular adsorption and/or interactions on various surfaces covering a quartz crystal. As a direct impact of mass adsorption, changes in frequency of the oscillating quartz crystal are detected (Dixon, 2008). An observed shift in the dissipation provides insights regarding the viscoelastic properties and rearrangements of the adlayer (Dixon, 2008).

### Critical RGD Motif Density for Binding Proteoliposomes

**FIGURE 2:**
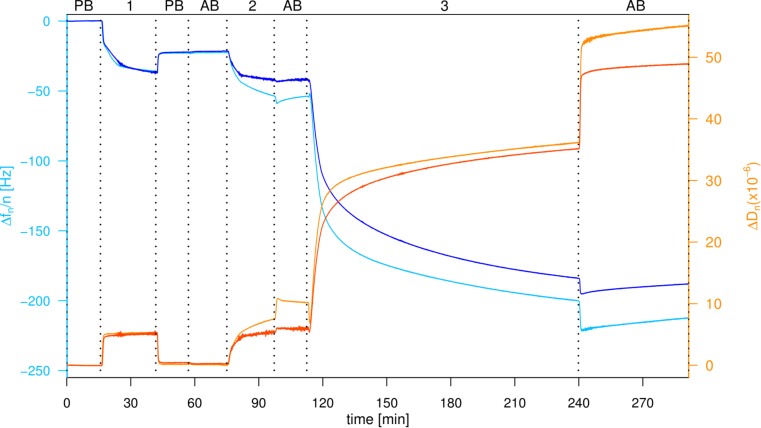
Attachment of proteoliposomes on an EG_3_OH/EG_6_RGD layer. QCM-D graph of the seventh overtone, frequency changes depicted in *blue*, dissipation changes in *orange*. Loading of the sensor with PB, passivation buffer; AB, activation buffer; 1:100 μM EG_3_OH/EG_6_RGD (60:40%), 2: Liposomes, 3: Proteoliposomes.

The high-density presentation of RGD was achieved with a straightforward and fast method by first conjugating RGD to EG_6_N_3_ via copper(I)-catalyzed azide-alkyne cycloaddition (CuAAC) and subsequently immobilizing on gold surfaces in the presence of EG_3_OH to adjust the density. As seen in the QCM-D profile, after proximately 30 min the decrease in frequency and increase in dissipation are stable, originating from adsorption of both OEG alkanethiols. After a subsequent washing step the frequency slightly increases and the dissipation decreases reaching a stable value indicating successful immobilization of a dense OEG layer on the gold surface (**Figure 2**). When the buffer was changed from passivation buffer to the same buffer containing 2 mM divalent ions (1 mM Mn^2+^ and 1 mM Mg^2+^, referred to as activation buffer) no change in frequency or dissipation was monitored. All further steps were performed in activation buffer since the affinity and specificity of integrins binding to the RGD motif is affected by the concentration of divalent ions (Zhang and Chen, 2012). As a control for unspecific attachment to the OEG and OEG-RGD layer, pure liposomes were washed over the modified surfaces. The frequency decreases again and the dissipation increases, indicating that liposomes adhere non-specifically and remain attached when washed with buffer. When proteoliposomes are washed over the modified surface, a frequency change as well as dissipation change is observed, which are even reinforced after a subsequent washing step. This observation indicates that the proteoliposomes stay intact and the integrins bind firmly to the RGD motif as they could not be removed by washing (**Figure 2**).

Based on this general experimental setup, the influence of the RGD motif density on the attachment of proteoliposomes was quantified. Modified surfaces were prepared with six different RGD motif densities ranging from 0 to 100% according to the adjustable well-defined system outlined above.

**FIGURE 3:**
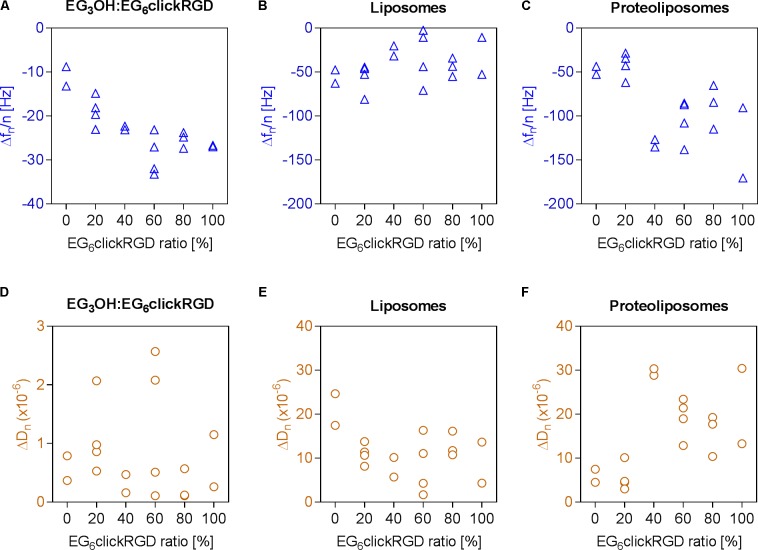
Adhesion of proteoliposomes to surfaces with varying RGD binding motif densities. **(A–C)** Frequency changes, **(D–F)** dissipation changes; **(A,D)** immobilization of OEG layer with EG_6_RGD ratios varying from 0 to 100%, **(B,E)** unspecific adhesion of pure liposomes, **(C,F)** subsequent adhesion of integrins reconstituted in liposomes to the RGD motif in the OEG layer.

In the initial SAM formation of the OEG layer, an increasing frequency change is observed at higher RGD motif densities. This correlation is effected by the amount of EG_6_RGD, which has a molecular weight of 1,120 g/mol and is consequently heavier than the EG_3_OH molecules (molecular weight = 671 g/mol). Dissipation changes, mainly between 0 × 10^−6^ and 1 × 10^−6^, indicate a rigid and well coupled OEG layer at all measured RGD densities (**Figures 3A,D**). Evaluation of the surface’s unspecific binding activity demonstrates that bare liposomes attach unspecifically to some extent to the surface, independently of the RGD ratio. Thereby, the frequency and dissipation changes for all RGD concentrations fluctuate around −41.82 ± 20.88 Hz and 11.28 × 10^−6^ ± 5.48 × 10^−6^, respectively (**Figures 3B,E**).

The subsequent attachment of proteoliposomes on the RGD motif functionalized surfaces was assessed in the next step. In general, decreasing frequency and dissipation values demonstrate a mass adsorption and formation of a viscoelastic adlayer, respectively. This indicates proteoliposome binding to the RGD motif on the surface via integrins. For an increased ratio of EG_6_RGD the graph shows biphasic curve characteristics (**Figures 3C,F**). In the first part, when the ratio of EG_6_RGD to EG_3_OH is 0 or 20% a frequency change of 43.98 ± 11.02 Hz and dissipation shift of 5.72 × 10^−6^ ± 2.37 × 10^−6^ occurs. Whereas in the second part, when the EG_6_RGD ratio exceeds or is equal to 40%, a higher frequency shift of −109.89 ± 30.94 Hz and simultaneously higher dissipation change of 20.79 × 10^−6^ ± 7.04 × 10^−6^ is detected. Based on these results an RGD motif density of 40% was employed for further experiments.

### Hyaluronan as Part of a Dual-Functionalized Surface Prevents Proteoliposome Binding

**FIGURE 4:**
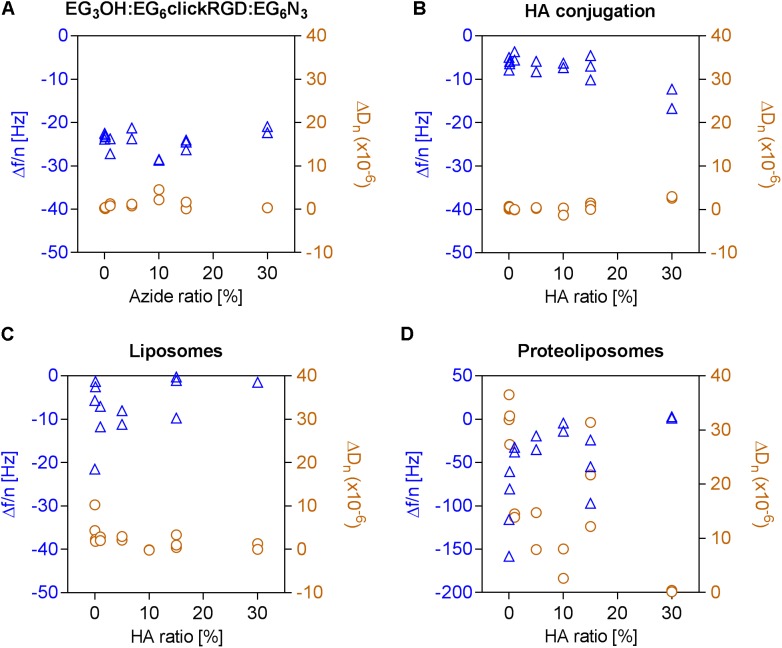
Adhesion of proteoliposomes to surfaces with a constant RGD motif density and varying ratios of conjugated HA. Frequency changes depicted in *blue triangles* and dissipation changes in *orange circles* were calculated based on the seventh overtone. **(A)** Immobilization of OEGs bearing an azide group (0–30%), **(B)** Followed by a conjugation of end-alkylated HA to the OEG layer by CuAAC, **(C)** Unspecific attachment of liposomes on the OEG layer, **(D)** Subsequent adhesion of proteoliposomes to the dual-functionalized surface.

In order to examine the adhesion of integrins in the presence of HA, dual-functionalized surfaces were prepared following the previously described procedure. This was achieved by conjugation of end-alkylated HA to an OEG layer, containing varying amounts of EG_6_N_3_ and EG_3_OH and a constant ratio of 40% EG_6_RGD. The immobilization of the OEG layers caused a constant frequency change, fluctuating around −24.25 ± 2.33 Hz, independently of the ratio of EG_6_N_3_ with general low dissipation values (0.96 × 10^−6^ ± 1.12 × 10^−6^). Directly after immobilization of the OEG layer, end-alkylated HA was conjugated to the EG_6_N_3_ in this OEG layer as indicated by the slight decrease in frequency and increase in dissipation, respectively (**Figures 4A,B**). Observation of the unspecific attachment of bare liposomes to these surfaces shows a constantly low level with a frequency change of −5.06 ± 6.36 Hz and dissipation change of 2.31 × 10^−6^ ± 2.49 × 10^−6^. These signal changes indicate that conjugated HA reduces the unspecific attachment of pure liposomes, which has been detected on the surfaces presenting the RGD motif alone (**Figure 4C**).

Finally, the effect of the dual-functionalized surface on the adhesion of integrins was investigated. It was observed that the frequency and dissipation changes caused by the binding of proteoliposomes declines with a higher EG_6_N_3_ content and consequently higher HA conjugation degree. At an initial EG_6_N_3_ ratio of 30%, only slight shifts in frequency and dissipation are detected indicating that the adhesion of proteoliposomes fails.

Even at lower ratios of HA between 0.01 and 30%, fewer proteoliposomes attach to the surface (**Figure 4D**). Thus, it can be concluded that minimal amounts of conjugated HA are sufficient to prevent the attachment of proteoliposomes to a surface with a constant RGD motif density. In this experiment, sHA molecules with a radius of gyration of around 5.3 nm were immobilized. Therefore, each conjugated HA molecule covers several molecules in the OEG layer including RGD binding motifs.

## Conclusion

Within this study a biomimetic model was established to address the challenge of co-presenting HA and the RGD motif to study integrin binding. The adhesion of α_IIb_β_3_ integrin, which was reconstituted in sphere-shaped lipid vesicles devoid of HA binding proteins, was quantified on HA and the RGD motif functionalized on an otherwise inert background. It is demonstrated that an RGD density of 40% has to be exceeded to achieve a prominent binding of proteoliposomes. Moreover, it is also concluded that even marginal amounts of HA are sufficient to significantly impact integrin attachment. The dissipation changes indicate that the proteoliposomes maintain an intact vesicle shape. Frohnmayer et al. (2015a,b) demonstrated that the adhesion of similarly prepared proteoliposomes on fibrinogen-coated surfaces, a protein which contains the RGD motif, results in decreased frequency and increased dissipation values within the same order of magnitude monitored in this study.

It would be especially beneficial to use these well-defined dual-functionalized surfaces to investigate the behavior of various cell lines in future studies. Especially, cancer cell lines are highly interesting since it is known that HA is highly enriched in their ECM. These cell-ECM interactions additionally involve binding of HA with receptors such as CD44 and RHAMM which are associated with tumor progression and metastasis (Liang et al., 2016; Morath et al., 2016; Rankin and Frankel, 2016).

## Data Availability

The raw data supporting the conclusions of this manuscript will be made available by the authors, without undue reservation, to any qualified researcher.

## Author Contributions

HB, BM, and CZ designed and conceived the experiments. BM performed preliminary experiments. CZ performed the experiments and analyzed the data. CZ and HB wrote the paper.

## Conflict of Interest Statement

The authors declare that the research was conducted in the absence of any commercial or financial relationships that could be construed as a potential conflict of interest.
